# The Correlation of Antibacterial Peptides Concentration in Umbilical Cord Blood and Early Onset Sepsis in Preterm Infants

**DOI:** 10.3389/fped.2022.903319

**Published:** 2022-05-19

**Authors:** Jiayu Miao, Zhuxiao Ren, Zhicheng Zhong, Fang Xu, Jianlan Wang, Jie Yang

**Affiliations:** ^1^Department of Pediatrics, Guangdong Women and Children Hospital, Guangzhou, China; ^2^Department of Neonatology, Guangdong Women and Children Hospital, Guangzhou, China; ^3^Department of Prenatal Diagnosis, Guangdong Women and Children Hospital, Guangzhou, China

**Keywords:** sepsis, antibacterial peptide, LL37, preterm infants, bio-maker

## Abstract

Umbilical cord blood from singleton preterm infants was collected during delivery, and the concentration of LL37 was measured. C-reactive protein (CRP), white blood cell count (WBC), platelets (PLT), and mean platelet volume (MPV) were determined within 3 days after birth. The differences in LL37, CRP, WBC, PLT, and MPV levels between the two groups were compared. Pearson correlation method was used to analyze the correlation between these factors. The early individual value of each detected index for early onset sepsis was analyzed by ROC curve. The level of LL37 in umbilical cord blood of sepsis group was significantly higher than those in the control group (383.85 ± 46.71 vs. 252.37 ± 83.30 ng/ml). Meanwhile, the levels of CRP, WBC, and MPV in the sepsis group were significantly higher than those in the control group (CRP:5.73 ± 4.19 vs. 2.50 ± 2.77 mg/L; WBC: 13.47 ± 12.35 vs. 6.83 ± 3.55 × 10^9^/L; MPV: 11.20 ± 1.11 vs. 8.90 ± 0.68 fL), the level of PLT was significantly lower than those in the control group (PLT: 161.00 ± 38.51 vs. 241.50 ± 49.85 × 10^9^/L) (*P* < 0.05). Pearson correlation analysis showed that the expression of LL37 was negatively correlated with PLT level (*r* = −0.9347, *P* < 0.0001), and positively correlated with MPV level (*r* = 0.9463, *P* < 0.0001). ROC curve analysis showed that the area under curve of LL37 for diagnosis of early onset sepsis was 0.875, the prediction probability was 0.7, the sensitivity was 90.0% and the specificity was 80.0%.

## Introduction

Neonatal sepsis is a systemic inflammatory response syndrome (SIRS) and is also the major cause of morbidity and mortality, especially in preterm infants (1). Early onset sepsis (EOS) has been defined based on the age at onset, as occurring in the first 3 days of life and is caused by bacterial pathogens transmitted vertically before or during delivery (2). Early recognition and diagnosis of early onset neonatal sepsis is required to prevent the transition into septic shock, which is associated with a mortality rate of at least 40% (3). Numerous studies have been conducted to assess the role of C-reactive protein (CRP), White blood cell (WBC), and other biomarkers for diagnosis of neonatal sepsis (4), but recently, there are some studies that report the important clinical value of antibacterial peptides for the diagnosis of sepsis (5).

The innate immune system is the first line of defense against microorganisms, of which cathelicidin family is one of the major antimicrobial peptide families in mammals, and LL-37 is the only known human cathelicidin (6). LL-37 exhibits diverse biological activities, including antimicrobial activity, anti-apoptosis, regulating inflammatory response and angiogenesis (7). We previously revealed that LL37 was one of the paracrine factors of umbilical cord blood mesenchymal stem cells and inhibited bacterial growth in ventilator -associated pneumonia (VAP) and sepsis (8, 9). Therefore, the level of LL-37 in umbilical cord blood may be able to influence the occurrence of early onset neonatal sepsis.

Platelets (PLT) have received significant concern for their role in the pathophysiology of infectious disease, thrombocytopenia is frequently observed following the onset of sepsis, and the decreased degree of PLT is closely related to the degree of SIRS. Platelets release cytokines and chemokines, recruit leukocytes, interact with bacteria and the endothelium, and contribute to microthrombi formation, which can become a pathological self-sustaining dysregulated process resulting in septic shock (10). Some markers of platelet function have been shown to correlate with severity of sepsis (11). Mean platelet volume (MPV) is a parameter of the average volume of peripheral blood platelets, which directly reflects the morphology of platelets, and is also a potential indicator for evaluating the pro-inflammatory state and thrombosis (12). However, few studies explore the predictive value of PLT and MPV for sepsis.

The aim of this study was to quantify the levels of antimicrobial peptides LL37 in umbilical cord blood, explore the predictability of LL37 for preterm infants at risk of early onset sepsis.

## Materials and Methods

### Study Population

This study was approved by the Ethics Committee of Guangdong Women and Children’s Hospital (201701026), registered at Clinical Trials.gov (NCT03053076). Written informed consent from mothers giving birth <33 weeks gestation (singleton) from 1 January 2019 to 30 June 2019 was obtained before collection of umbilical cord blood samples. The excluded criteria were as follows: (1) Mothers with a history of infection (virus infection, clinical chorioamnionitis) before delivery and premature rupture of membranes; (2) Preterm infants with congenital abnormalities, meconium pollution of amniotic fluid. If the neonates fulfilled the included criteria, we would talk with their parents for collection of umbilical cord blood; all of them who were diagnosed as early onset sepsis would be assigned to sepsis group and other neonates without early onset sepsis were assigned to control group (matched by 1:2 according to gestational age and sex). Umbilical cord blood was sampled during delivery using a standardized procedure. Placentae were examined for evidence of histological chorioamnionitis. Clinical data of maternal and preterm infants were collected.

### Measurement of Concentrations of Antimicrobial Peptides LL37

The level of LL37 in umbilical cord blood was measured by sandwich ELISA as follows: The micro ELISA plate provided in this kit has been pre-coated with an antibody specific to Human LL-37. Standards or samples are added to the micro ELISA plate wells and combined with the specific antibody. Then a biotinylated detection antibody specific for Human LL-37 and Avidin-Horseradish Peroxidase (HRP) conjugate are added successively to each micro plate well and incubated. Free components are washed away. The substrate solution is added to each well. Only those wells that contain Human LL-37, biotinylated detection antibody and Avidin-HRP conjugate will appear blue in color. The enzyme-substrate reaction is terminated by the addition of stop solution and the color turns yellow. The optical density (OD) is measured spectrophotometrically at a wavelength of 450 ± 2 nm. The OD value is proportional to the concentration of Human LL-37.

### Measurement of Inflammatory Mediators

The concentration of inflammatory mediators within 3 days after birth was measured. The concentration of CRP in venous blood was measured by routine latex-enhanced immunoturbidimetry (Roche Diagnostics). Routine WBC was determined by flow cytometry (Sysmex). The PLT and MPV were measured by whole blood cell automatic analyzer (Japanese Sysmex KX221). The average concentration of inflammatory mediators was analyzed.

### Statistical Analysis

The normal distribution data was represented as the mean ± standard deviation, analyzed by *T*-tests. The data which was not normally distributed was represented as the median ± standard deviation, analyzed by non-parametric tests. Dichotomous data was expressed by the frequency and relevant percentage, analyzed by Chi-square test. The correlation between the measurement data was analyzed by Pearson correlation. Receiver operating characteristic curve (ROC) was applied to evaluate the ability of antibacterial peptide LL-37 and related inflammation indicators to diagnose early onset sepsis, and analyze its sensitivity and specificity. *P* < 0.05 was considered statistically significant. All analyses were performed by using SPSS version 19.0 software. All figures were performed by GraphPad Prism 5.

## Results

### General Characteristics

**FIGURE 1:**
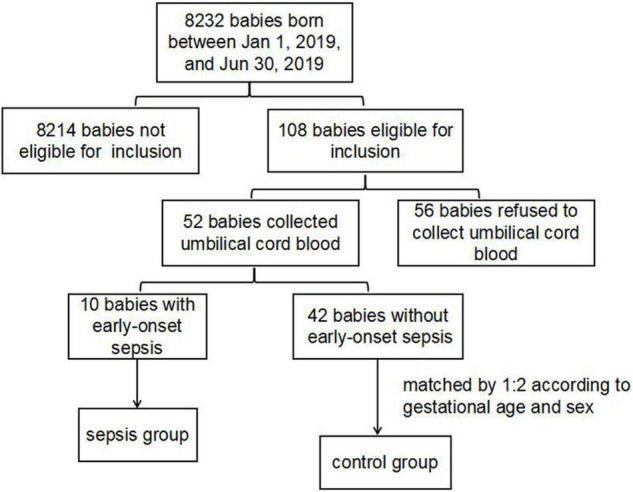
Participant flow for the primary analysis.

There were a total of 8,232 newborns delivered in Guangdong Women and Children Hospital from 1 January 2019 to 30 June 2019. A total of 108 newborns were eligible for inclusion. Fifty-two of them agreed to collect umbilical cord blood and others refused. There were ten preterm infants of 52 in early onset sepsis group, and twenty preterm infants were enrolled as control group matched by 1:2 according to gestational age and sex (as shown in Figure 1). There were no significant differences in maternal age, prenatal hormones, prenatal antibiotics, histological chorioamnionitis, diabetes, eclampsia in two groups. In the sepsis group, the occurrence of neonatal septic shock was significantly higher than that in control group, the difference was statistically significant (*P* = 0.030). But there were no significant differences in sex, gestational age, birth weight, delivery method, and other infectious disease like meningitis and pneumonia in the two groups (*P* > 0.05). Patient characteristics are summarized in Table 1.

**TABLE 1:** Maternal and neonatal characteristics.

	Sepsis group (*n* = 10)	Control group (*n* = 20)	*P*
**Maternal**			
Age (y)	32.30 ± 6.25	31.10 ± 5.86	0.609
Prenatal glucocorticoids, *n*, (%)	7 (70.0%)	18 (90.0%)	0.300
Prenatal antibiotics, *n*, (%)	7 (70.0%)	8 (40.0%)	0.245
Diabetes, *n*, (%)	5 (50%)	3 (15%)	0.078
Eclampsia, *n*, (%)	1 (10%)	3 (15%)	1.000
**Neonatal**			
Male, *n*, (%)	8 (80%)	16 (80%)	1.000
Gestational age (w)	29.73 ± 1.94	30.39 ± 1.61	0.336
Birth weight (kg)	1.27 ± 0.32	1.46 ± 0.23	0.071
Cesarean section, *n*, (%)	4 (40.0%)	10 (50.0%)	0.709
Septic shock, *n*, (%)	3 (30%)	0 (0%)	0.030
Meningitis, *n*, (%)	2 (20%)	0 (0%)	0.103

*Data are expressed as n (%) or mean ± standard deviation. P < 0.05, the difference was statistically significant.*

### The Levels of LL37 and Other Inflammatory Mediators

**FIGURE 2:**
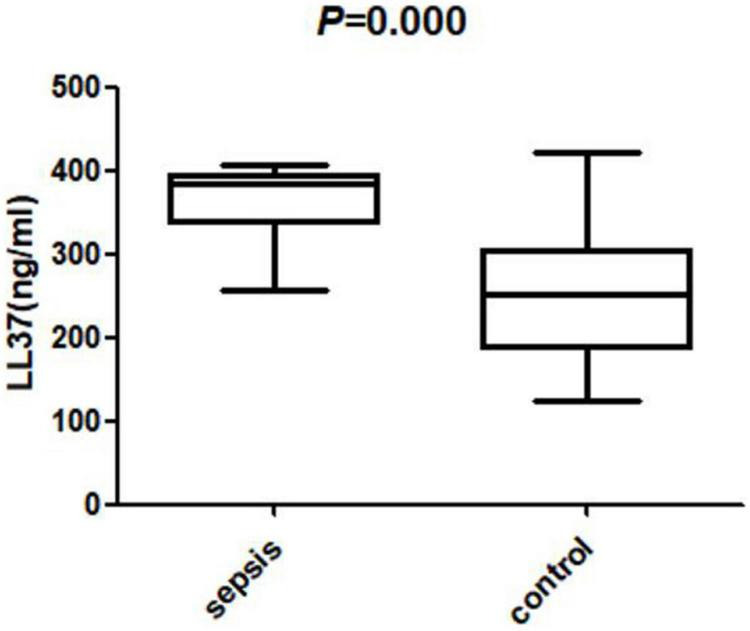
The level of LL37 in umbilical cord blood of two groups.

**FIGURE 3:**
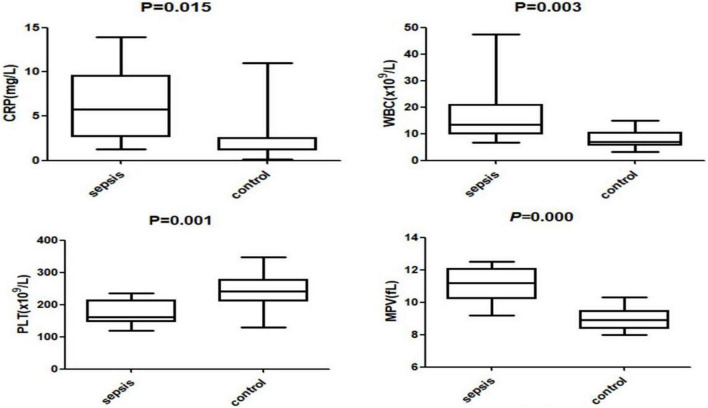
The levels of C-reactive protein (CRP), white blood cell count (WBC), platelet (PLT) and mean platelet volume (MPV) in the two groups.

The levels of LL37 in umbilical cord blood of sepsis group were significantly higher compared with controls (383.85 ± 46.71 vs. 252.37 ± 83.30 ng/ml). The levels of CRP, WBC, and MPV in sepsis group were also higher than those in the control group (CRP:5.73 ± 4.19 vs. 2.50 ± 2.77 mg/L; WBC: 13.47 ± 12.35 vs. 6.83 ± 3.55 × 109/L; MPV: 11.20 ± 1.11 vs. 8.90 ± 0.68 fL), the levels of PLT in the sepsis group were significantly lower (161.00 ± 38.51 vs. 241.50 ± 49.85 × 109/L), all of the difference above was statistically significant (*P* < 0.05) (Figures 2, 3).

### Correlation Between Levels of LL37 and Mediators of Inflammation

**FIGURE 4:**
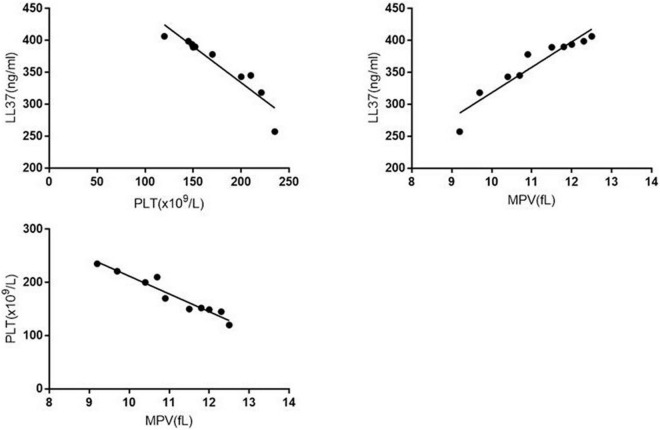
The correlation of LL37, PLT, and MPV in sepsis group.

In the sepsis group, the LL37 levels correlated with the levels of PLT (*P* < 0.0001, *r* = −0.9347), MPV (*P* < 0.0001, *r* = 0.9463). The levels of PLT correlated with the levels of MPV (*P* < 0.0001, *r* = −0.9641). No significant correlation was found in the control group (Figure 4).

### The Analysis of Receiver Operating Characteristic Curve

**FIGURE 5:**
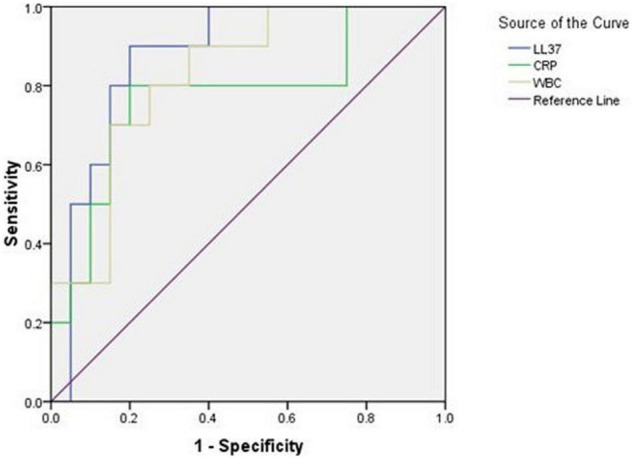
Receiver operating characteristic (ROC) curve of LL37, CRP, and WBC for early diagnosis of early onset sepsis.

Antimicrobial peptides LL-37, CRP, and WBC all have early diagnostic value for early onset neonatal sepsis. However, the area under the ROC curve (AUC) diagnosed with LL37 is the largest (LL37:0.875, CRP:0.775, and WBC:0.825), and when the prediction probability of LL37 was 0.7, the sensitivity was 90.0%, and the specificity was 80.0% (Figure 5 and Table 2).

**TABLE 2:** The diagnostic value of LL37, CRP, and WBC for early onset neonatal sepsis.

Index	AUC (95% CI)	Cutoff	Sensitivity (%)	Specificity (%)
LL37	0.875 (0.747–1.003)	0.70	90.0	80.0
CRP	0.775 (0.581–0.969)	0.60	80.0	80.0
WBC	0.825 (0.674–0.976)	0.55	80.0	75.0

*CRP, C-reactive protein; WBC, white blood cells; AUC, the area under the receiver operating characteristic curve; 95% CI, 95% confidence interval.*

## Discussion

### Principal Findings

Neonatal sepsis is the major cause of prematurity and infant mortality. Early diagnosis and effective treatment are keys to save the lives of neonates. In this prospective study, we determined the concentrations of LL37 in umbilical cord blood and other inflammatory factors in peripheral blood in preterm infants. The key findings of this study are the following: (1) The preterm infants at risk of early onset sepsis had higher levels of antimicrobial peptide LL37, CRP, WBC, and MPV, and a lower level of PLT. (2) The expression of antimicrobial peptide LL37 was negatively correlated with PLT level and positively correlated with MPV level, the expression of PLT was negatively correlated with MPV level in preterm infants at risk of early onset sepsis. (3) The early individual value of antimicrobial peptide LL37 for early onset sepsis is higher than CRP and WBC.

### Strengths and Weaknesses

This study has strengths as follows: First, we measured the concentrations of LL37 in umbilical cord blood in preterm infants, analyzed the relationship between LL37, and neonatal early onset sepsis, which was not reported previously. LL-37 is released as an 18-kDa pro-peptide, produced by hCAP-18, which is significantly increased in inflammation and infection, has the function of amplifying Toll-like receptor signals (13, 14). Bucki et al. confirmed that LL-37 rose immediately after infection, and reached a peak within 2 h, which provided a theoretical basis for early diagnosis of sepsis (15). Second, in our study, the expression of antimicrobial peptide LL37 was negatively correlated with PLT level and positively correlated with MPV level in preterm infants at risk of early onset sepsis. LL-37 has been shown to inhibit P-selectin expression, which is a major platelet alpha-granule protein that is highly expressed on the platelet surface during activation (16). However, P-selectin can lead activated platelets adhere to neutrophils and monocytes (17), with innate immune response of LL37. There may be a link between the anti-inflammation activity of LL-37 and its antiplatelet aggregation activity. However, this study also has some limitations. First, the number of early onset sepsis cases was limited and the efficacy needed to expand the sample size for further evaluation. Second, LPS activates inflammatory cells to secrete TNF-a, IL-1β, IL-6 and other cytokines, which mediate the development of sepsis (18). LL37 was proved to be able to bind to LPS and suppress the interaction between LPS and LPS binding protein as well as bind to macrophage CD14 receptors, thus inhibiting LPS-induced TNF-a expression (19, 20). We may conduct a larger study to assess the mechanism of LL37 and sepsis. Third, the study found the correlation of LL37 with PLT and MPV. However, we didn’t know specific signals or pathways that LL-37 influenced. Wen et al. reported that LL-37 also could reduce phosphorylation of Src kinase and AktSer^473^, indicating that the modulation of Src/PI3k/Akt signaling pathway involved in the antiplatelet activity of LL-37 (16). Src kinase plays an important role in activating platelet (21), and phosphatidylinositol 3-kinase (PI3K) is the most crucial one among the downstream effectors of Src kinase, Akt is the most well-known activation marker of PI3K. Therefore, we may conduct an animal study to explore how the LL37 influence platelet in early onset neonatal sepsis. Fourth, there was a study found that the levels of LL-37, PCT, and CRP in peripheral blood of sepsis group were significantly higher than those of healthy control group, and the expression of LL-37 was positively correlated with PCT and CRP levels, but the value of combined diagnosis of sepsis was greater (22). Due to the limited cases, we could not do a combined prediction. Finally, we ignored the influence of prenatal and perinatal factors to the concentration of LL37, such as prematurity, use of glucocorticoids, prolonged labor or perinatal asphyxia and so on. So, in an ongoing study, we would compare the umbilical cord blood level of LL37 between term infants and preterm infants, and also the level of LL37 between the umbilical cord blood and serum, more new discoveries would be reported in follow-up articles.

### Comparison With Previous Studies

In the present studies, it is not surprising to find significant elevation in the serum level of CRP, PCT, and WBC in the newborn with sepsis. CRP is the most common laboratory tests in the diagnosis of neonatal sepsis (23), which will take around 10 to 12 h for level to increase, so making it low sensitive for early diagnosis of neonatal sepsis (24). As well as this study provides low sensitivity (80.0%) with limited specificity (80.0%) for CRP. Meanwhile, conditions where there is spurious increase in level of CRP such as meconium aspiration syndrome (MAS), premature infant exposure to glucocorticoids, maternal fever during labor, fetal distress, prolonged labor, perinatal asphyxia, and intraventricular hemorrhage (IVH), thus making it a non-specific biomarker for diagnosis of neonatal sepsis (24, 25).

Leukopenia has been reported to have low sensitivity (29%) but high specificity (91%) for diagnosis of neonatal sepsis (26), which is defined as WBC count less than 5,000 to 7,500/mm3. But in our study, there is a different sensitivity (80.0%) and specificity (75.0%). Besides WBC, absolute neutrophil count (ANC), and immature to total leukocyte ratio (I:T) also have been used to diagnosis of neonatal sepsis. Hornik et al. found that low WBC counts, low ANC and high I: T neutrophil ratios were associated with increased odds of infection (27). But all have significant limitations in the diagnosis of neonatal sepsis.

Procalcitonin (PCT) is an acute phase reactant protein and has been reported to be associated with immunomodulation associated with SIRS. The levels of PCT increase significantly during systemic bacterial infection such as early onset or late-onset sepsis (28, 29), and Chiesa et al. also showed in diagnosis of EOS, PCT had sensitivity of 92%, specificity of 97% (29). However, non-specific elevation of PCT levels in the absence of bacterial infection can occur in situations of massive stress, such as premature newborn, intracranial hemorrhage, birth asphyxia, neonatal hypoxemia, and healthy infants born to mothers with chorioamnionitis (30). Therefore, PCT must be studied further in larger groups of infants so as to improve its diagnostic accuracy. As there are limited cases in our study, PCT showed no statistical difference in two groups, and without increasing significantly in early onset sepsis.

Studies have confirmed that LL-37 rises immediately after infection and reaches a peak within 2 h (31). Therefore, the serum levels of LL-37 are a useful marker in the diagnosis of neonatal sepsis. El-Salam et al. found the serum levels of CRP and LL-37 were significantly higher in the newborn with clinical sepsis than healthy newborn, and LL37 had an advantage in the diagnosis of early onset sepsis over than CRP (32). Similar findings were reported by Gallo et al. (33) and Peter et al. (34) who showed that production of LL37 was increased adaptively in response to a number of specific infections, contributed to innate host defenses by mediating recruitment and enhanced survival of neutrophils and apoptosis of infected epithelial cells. With this study, we investigated the levels of LL37 in umbilical cord blood and found that it was significantly higher in preterm with early onset sepsis than that in healthy control group. The sensitivity and specificity of LL37 were 90.0 and 80.0%, respectively, values that were higher than the most obtained for the other markers. The inflammatory mediators (interleukin IL-6, IL-1, tumor necrosis factor TNF) released after severe infection, activating the IF-IL-6 site on the antimicrobial peptide LL-37 by Kinase/signal and transcription activator (JAK/STAT) pathway or Ras-dependent mitogen-activated protein kinase (MAPK) pathway, so significantly increasing the expression of LL-37 (35). This may be the main mechanism.

## Conclusion

In summary, the expression of antimicrobial peptide LL-37 increased significantly in umbilical cord blood in preterm at a risk of early onset sepsis, which has certain clinical application value for early diagnosis and evaluation of sepsis, and is expected to become a new index for clinical prediction of severe infection screening.

## Data Availability Statement

The raw data supporting the conclusions of this article will be made available by the authors, without undue reservation.

## Ethics Statement

This study was approved by the Ethics Committee of Guangdong Women and Children’s Hospital (201801065).

## Author Contributions

JM collected all data and wrote the manuscript. ZR and ZZ collected the samples. JW collected the references. FX and JY revised and reported the manuscript. All authors read and approved the final manuscript.

## Conflict of Interest

The authors declare that the research was conducted in the absence of any commercial or financial relationships that could be construed as a potential conflict of interest.

## Publisher’s Note

All claims expressed in this article are solely those of the authors and do not necessarily represent those of their affiliated organizations, or those of the publisher, the editors and the reviewers. Any product that may be evaluated in this article, or claim that may be made by its manufacturer, is not guaranteed or endorsed by the publisher.
